# A novel *DSPP *mutation causes dentinogenesis imperfecta type II in a large Mongolian family

**DOI:** 10.1186/1471-2350-11-23

**Published:** 2010-02-10

**Authors:** Haihua Bai, Hasi Agula, Qizhu Wu, Wenyu Zhou, Yujing Sun, Yue Qi, Suya Latu, Yujie Chen, Jiri Mutu, Changchun Qiu

**Affiliations:** 1 Inner Mongolia University, Huhhot 010021, China; 2 Inner Mongolia University for the Nationalities, Tongliao 028000, China; 3 National Laboratory of Medical Molecular Biology, Institute of Basic Medical Sciences, Chinese Academy of Medical Sciences/Peking Union Medical College (CAMS/PUMC), 5 Dong Dan San Tiao, Beijing 100005, China

## Abstract

**Background:**

Several studies have shown that the clinical phenotypes of dentinogenesis imperfecta type II (DGI-II) may be caused by mutations in *dentin sialophosphoprotein *(*DSPP*). However, no previous studies have documented the clinical phenotype and genetic basis of DGI-II in a Mongolian family from China.

**Methods:**

We identified a large five-generation Mongolian family from China with DGI-II, comprising 64 living family members of whom 22 were affected. Linkage analysis of five polymorphic markers flanking *DSPP *gene was used to genotype the families and to construct the haplotypes of these families. All five DSPP exons including the intron-exon boundaries were PCR-amplified and sequenced in 48 members of this large family.

**Results:**

All affected individuals showed discoloration and severe attrition of their teeth, with obliterated pulp chambers and without progressive high frequency hearing loss or skeletal abnormalities. No recombination was found at five polymorphic markers flanking DSPP in the family. Direct DNA sequencing identified a novel A→G transition mutation adjacent to the donor splicing site within intron 3 in all affected individuals but not in the unaffected family members and 50 unrelated Mongolian individuals.

**Conclusion:**

This study identified a novel mutation (IVS3+3A→G) in *DSPP*, which caused DGI-II in a large Mongolian family. This expands the spectrum of mutations leading to DGI-II.

## Background

Dentinogenesis imperfecta type II (DGI-II) (OMIM # 125490) is an autosomal dominant dental disorder with a complete penetrance that affects both the primary and the permanent teeth [1]. DGI-II is characterized by amber and opalescent teeth, abnormal dentine leading to obliteration of the pulp chamber, and enamel that, although unaffected, tends to fracture. This causes the dentine to undergo rapid attrition, leading to a marked shortening of the teeth. The gene DSPP is located in the 6.6-cM D4S2691-D4S2692 interval at 4q21 and encodes a precursor protein, which is cleaved to yield dentine sialoprotein (DSP) and dentine phosphoprotein (DPP) [2-4]. A nonsense mutation in DSPP has been reported to cause DGI-II in a Chinese family [5] and other DSPP mutations have subsequently been demonstrated in Chinese families with DGI-II [6-9]. In addition, families with DGI-II in other countries have been reported with mutations in *DSPP *[10-15]. However, the genetic basis of DGI-II in Mongolian families has not been explored before. In the present study, we describe a large, five-generation Mongolian family with DGI-II and report a novel DSPP mutation in this family.

## Methods

### Patients

**Figure 1:**
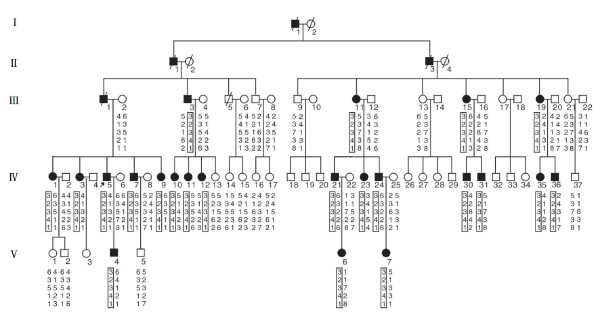
**Pedigree structure of a Mongolian family affected by dentinogenesis imperfecta type II (DGI-II)**. Affected males and females are indicated by filled squares and circles, respectively. Normal individuals are shown as empty symbols. The proband is IV5. Two-point linkage analysis was conducted using five polymorphic microsatellite markers (*GATA62A11, D3S564, D4S1317, D4S3132 and D4S1563*) in region 4q21.3. Genotype results are shown under each symbol. Note that haplotype 3-2-3-4-1 co-segregates with affected individuals, suggesting linkage of *DSPP *to DGI-II in this family.

We identified a large, five-generation Mongolian family with DGI-II consisting of 64 living family members, of which 22 were affected (Figure 1). All living members were examined clinically and taken for panoramic dental tomograms. The clinical and radiographic images were published under the patients' written permission. The study "Gene Research on Dentinogenesis Imperfecta in Mongolian Families" was approved by the Research Ethics Committee of Peking Union Medical College.

### DNA extraction

Peripheral blood leukocytes were collected from 48 of the 64 family members, and human genomic DNA was extracted by using phenol - chloroform followed by ethanol precipitation.

### Genetic linkage and haplotype analysis

Two-point linkage analysis was conducted using five polymorphic markers (*GATA62A11*, *D3S564*, *D4S1317*, *D4S3132 *and *D4S1563*) at 4q21.3. LOD scores were calculated using the MLINK program of the LINKAGE package. The parameters used for linkage analysis were autosomal dominant inheritance, complete penetrance, a mutation rate of zero, equal male-female recombination rates, equal allele frequency, and a disease allele frequency of 1 in 10,000.

### Sequence analysis of DSPP

Mutation screening was carried out using direct DNA sequence analysis. The exons of the *DSPP *gene were amplified by primers flanking the exon-intron boundaries (Table 1). Exon 4 was amplified into two, and Exon 5 was amplified into six fragments. PCR conditions for exons 1-5 were as followlling: a 5-min initial denaturation at 94°C, 35 cycles of 1- min denaturation at 94°C, 1- min annealing at 58°C, 58°C,50°C, 60°C, 60°C, 60°C,64°C, 60°C, 60°C, 55°C, and 55°C, respectively, and a 1-min extension at 72°C, and a 5-min final extension at 72°C. PCR product were sequenced by Beijing AuGCT Biotechnology Co., Ltd http://http:www.augct.com.

**Table 1:** Primers used for amplification and sequencing of the DSPP gene

Exon	Forward primer sequence (5'--3')	Reverse primer sequence (5'--3')	Annealing Temperature (°C)
1	TCACCAAGTGAAGGAAGTGG	AAAGCCCAAGGTGGATTTTT	58°C
2	GATGCCCCCATAACCACACC	CTCCATGACTTCTGGGCATT	58°C
3	AAGAACCTTTTCAATAGCCAGT	TGGAGAAGTTAATGGAATGTAGCAAC	50°C
4-1	TGCAATTTGCTTTCCTTCAAG	TGTTATTGCTTCCAGCTACTTGAG	60°C
4-2	CAATGAGGATGTCGCTGTTG	TGCCATTGAAAGAAATCAGC	60°C
5-1	TTCTTTCCTCCATCCTTCCATAG	TGTCATCATTCCCATTGTTACC	60°C
5-2	CAAAAGGAGCAGAAGATGATGAC	TTGCTGCTGTCTGACTTGCT	64°C
5-3	CAAATCAGACAGTGGCAAAGGTAAAT	CACTGCTATTGCTGCTGTCGTTGCT	60°C
5-4	GACAGCAGTAATAGTAACAGCAGCG	GCTGTCGCTGCTATTGCTATCACTG	60°C
5-5	GCAGTGACAGCAACGAAAGCAGCAAT	GTTGTTACCGTTACCAGACTTGCTC	55°C
5-6	TGACAGCACATCTGACAGCAAT	TCCCCCAGTTGTTTTTGTTT	55°C

We determined the sequences of all five exons and the exon flanking sequences of *DSPP *from 48 of affected and unaffected individuals in this family. The mutaton sites of 50 unrelated healthy Mongolian controls also were sequenced directly.

### Prediction of the mutation effect

In order to investigate whether the mutation will affect the splice donor site of exon 3, we used the BDGP site http://www.fruitfly.org/seq_tools/splice.html to predicte the effect of gene mutation on the splicing site of the DSPP[16].

## Results

**Figure 2:**
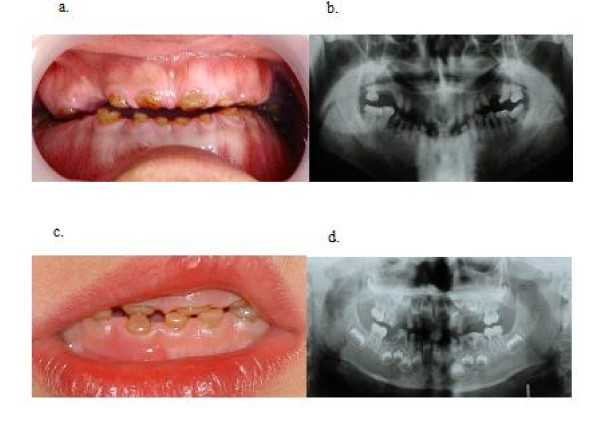
**Clinical analysis of dentinogenesis imperfecta type II (DGI-II)**. The proband (IV5) is a man aged 32. His permanent teeth showed a shade of brown and almost complete attrition of the enamel layer without a history of periapical infections(a and b). Dentition of the 5-year-old son of the proband. His primary teeth had shown normal timing of eruption, but shortly thereafter become brownish and small due to cracking of the enamel and attrition of dentin. At the time of examination, his first permanent molars had just emerged and still showed an intact enamel(c and d).

In the five-generation Mongolian family with DGI-II, the proband was a man aged 32 (Figure 1, IV5 ). His permanent teeth showed a shade of brown and almost complete attrition of the enamel layer without a history of periapical infections. All affected individuals showed discoloration and severe attrition of their teeth with obliterated pulp chambers. In addition, the enamel, although unaffected, had tended to fracture, causing the dentine to undergo rapid attrition, leading to a marked shortening of the teeth. Both the primary and the permanent teeth were affected (Figure 2). No high-frequency hearing loss or obvious skeletal abnormalities were found in any of the affected individuals.

Through linkage analysis we obtained a maximal LOD score of 6.06 for marker D3S564 at θ = 0.00, thereby demonstrating definitive linkage. Haplotype analysis showed that haplotype 3-2-3-4-1 cosegregated with the disease in this family, indicating that the disease locus was linked to the chromosome region harboring *DSPP*, and that *DSPP *was a candidate gene (Figure 1).

**Figure 3:**
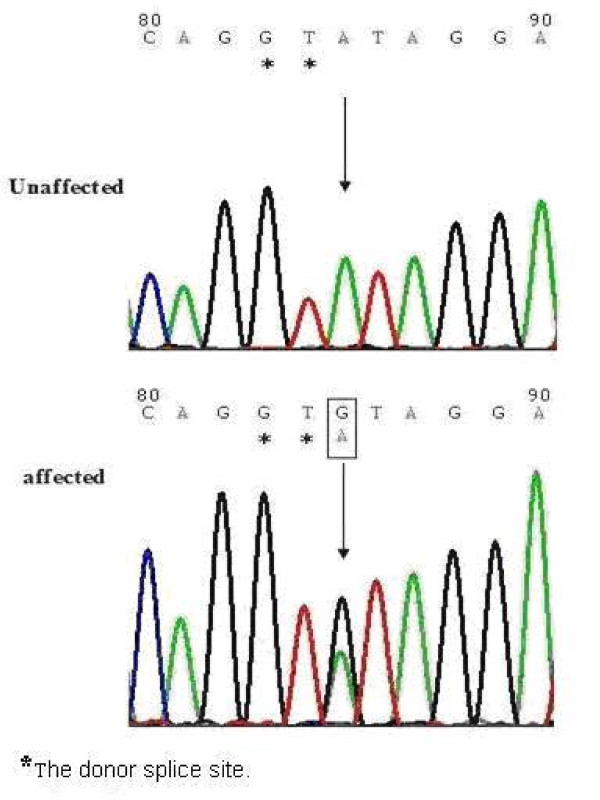
**Identification of a novel mutation**. An A→G transition adjacent to the donor splicing site (GT) within intron 3 of *DSPP *was detected in all affected individuals, whereas this mutation was not detected in unaffected individuals of the DGI-II Mongolian family or in unrelated healthy Mongolian controls. DNA sequences for a normal family member (upper panel) and the proband IV5 (lower panel).

Mutation screening showed a novel, functional A→G transition mutation adjacent to the donor splicing site (GT) within intron 3 of *DSPP *in all affected individuals, whereas this mutation was not found among the unaffected individuals in the family (Figure 3). Furthermore, we did not find this mutation in 50 unrelated, healthy Mongolian controls.

The available splicing site prediction software, the BDGP site, was utilized to predict the consequence of the mutation (IVS3+3A→G) in *DSPP*, the splice donor site of exon3 went from a score of 0.89 to <0.

## Discussion

We identified a novel mutation (IVS3+3A→G) in *DSPP *in a large Mongolian family suffering from dentinogenesis imperfecta II (DGI-II). This novel mutation (IVS3+3A→G) resulted in a donor splicing site change from wild-type GTAT to mutated GTGT in one of the two *DSPP *alleles that co-segregate in affected individuals. This mutation did not exist in unaffected family members or in an additional 50 healthy Mongolian controls. These results suggest that the A→G mutation caused DGI-II in this Mongolian family.

DGI-II is a clinically heterogeneous disorder caused by *DSPP *mutations [7,17-19]. Previous studies have reported DGI-II families with a mis-sense mutation in exon 2 [6], a nonsense mutation in exon 3 [5], splicing site mutations in intron 3+1 [6,9] and a frameshift mutation in intron 2 [9]. However, the molecular mechanisms by which *DSPP *mutations cause DGI-II are still unclear. In this Mongolian family, we speculate that the novel mutation is likely to produce a new splicing site and destroy the original splicing site within intron 3. This mutation may result in the abnormal intron splicing and lead to exon-skipping with a loss of exon 3, which encodes part of dentin sialoprotein protein. Because tissue samples from this family were unavailable, we were unable to prepare mRNA from the affected individuals to determine the sequences of *DSPP *transcripts.

To our knowledge, this study is the first report of a novel *DSPP *mutation causing DGI-II in a Mongolian family from China. This mutation differs from those found previously in other Chinese families and in families of other ethnic groups. Mongolians represent one of the major ethnic minority groups in China. They reside on the Inner Mongolian grassland in the northeast of China, where they live a nomadic lifestyle. This Mongolian family, whose forebears lived on the Horqin grassland in the eastern part of Inner Mongolia for many generations, is a relatively homogeneous population with characteristics that are advantageous for genetic research, including low divorce rate, limited mobility, consistent dietary habits and favorable environmental factors.

## Conclusion

This study documents a novel A→G transition mutation adjacent to the donor splicing site (GT) within intron 3 of *DSPP *that causes DGI-II in a large Mongolian family. This expands the spectrum of mutations that cause DGI-II.

## Abbreviations

DGI: dentinogenesis imperfecta; DPP: dentine phosphoprotein; DSP: dentine sialoprotein; DSPP: dentin sialophosphoprotein

## Competing interests

The authors declare that they have no competing interests.

## Authors' contributions

HB designed the study and family recruitment, performed the linkage analysis, drafted the manuscript and obtained funding. HA supervised the study design. QW conducted clinical diagnoses and family recruitment. JM supervised the study and family recruitment. CQ did the genetic design, supervised the study and obtained funding. All other authors provided technical assistance, and all authors read and approved the final manuscript.

## Pre-publication history

The pre-publication history for this paper can be accessed here:

http://www.biomedcentral.com/1471-2350/11/23/prepub
